# Characteristics of unvaccinated and vaccinated critically ill COVID-19 patients in calabria region (Italy): A retrospective study

**DOI:** 10.3389/fmed.2022.1042411

**Published:** 2022-11-24

**Authors:** Andrea Bruni, Federico Longhini, Sebastiano Macheda, Eugenio Biamonte, Pino Pasqua, Giuseppe Neri, Maria Laura Guzzo, Eugenio Garofalo, Calabria COVID-ICU Network authors, Antonio Caroleo

**Affiliations:** Anesthesia and Intensive Care, Department of Medical and Surgical Sciences, “Magna Graecia” University, Catanzaro, Italy; Anesthesia and Intensive Care Unit, Grande Ospedale Metropolitano, Reggio Calabria, Italy; Anesthesia and Intensive Care Unit, Annunziata Hospital, Cosenza, Italy; Anesthesia and Intensive Care Unit, “Pugliese Ciaccio” Hospital, Catanzaro, Italy; ^1^Anaesthesia and Intensive Care, Department of Medical and Surgical Sciences, Magna Græcia University, Catanzaro, Italy; ^2^Anesthesia and Intensive Care Unit, Grande Ospedale Metropolitano, Reggio Calabria, Italy; ^3^Anesthesia and Intensive Care Unit, Annunziata Hospital, Cosenza, Italy; ^4^Anesthesia and Intensive Care Unit, Hospital Pugliese Ciaccio, Catanzaro, Italy

**Keywords:** COVID-19, vaccination, critical care, acute respiratory failure, gas exchange, booster vaccination

## Abstract

**Introduction:**

After the rapid surge of a novel coronavirus (SARS-CoV-2) in 2020 anti-SARS-CoV-2 vaccines have been developed to prevent the development of critical forms of COVID-19 leading to Intensive Care Unit (ICU) admission. The possibility of ICU admission after the first-cycle vaccination has been already reported; however, no data have been published regarding vaccinated patients with a “booster” dose. This retrospective study describes the characteristics of critically ill patients after the implementation of the regional “booster” dose vaccination program in a southern region of Italy.

**Materials and methods:**

We screened all medical records of critically ill COVID-19 patients in the period between January to April 2022. We collected the demographic characteristics, the presence of comorbidities, the vaccination status, the clinical course (arterial blood gases and type of respiratory support) and outcomes (rate of tracheostomy, ICU length of stay and mortality).

**Results:**

A total of 272 patients were admitted to ICUs during the study period. 161 patients were unvaccinated, whereas 111 were vaccinated with the complete first-cycle or “booster” dose. The type of respiratory support was similar between groups. Vaccinated patients were characterized by a better oxygenation throughout the whole ICU length of stay. Fourteen unvaccinated and 3 vaccinated patients required tracheostomy (*p* = 0.045). ICU length of stay was 12.2 (± 7.3) days in unvaccinated patients and 10.4 (± 6.7) days in vaccinated patients (*p* = 0.036). ICU mortalities were 38.5 and 24.3% in unvaccinated and vaccinated patients, respectively (*p* = 0.014).

**Conclusion:**

Vaccinated patients have better clinical course and outcomes as compared to the unvaccinated population.

## Introduction

The rapid surge of a novel coronavirus (SARS-CoV-2) in 2020 exceeded the capacity of Intensive Care Units (ICU) to care patients with acute respiratory failure and SARS-CoV-2 related disease (COVID-19) (1–3). Behind the attempt to test several pharmacological therapies to treat patients with COVID-19 (4–7), anti-SARS-CoV-2 vaccines have been developed to prevent the development of critical forms of COVID-19 leading to ICU admission (8). The first cycle of vaccination with BNT162b2 vaccine consists in two consecutives mRNA doses administered 21 days apart (9),and it induces an efficient immune response in 95% of naïve individuals (10). However, the effect of the first cycle with anti-SARS-CoV-2 vaccine wanes over a period of months, deeming necessary a “booster” dose (8, 11, 12), particularly in immunosuppressed patients (13). Indeed, a cohort study conducted in a northern region of Italy has reported that 18% of patients admitted in the ICU received the complete first vaccination cycle since a median time of 5 to 6 months, while 8% were partially vaccinated and waiting for the second dose (14). However, in patients with advanced age or comorbidities, critical COVID-19 has been reported despite recent vaccination (15, 16).

We have therefore designed this observational study to describe the characteristics of critically ill patients admitted during the 4th waves of COVID-19 patients, after the implementation of the regional vaccination program for the “booster” dose (September 2021) in Calabria, a southern region of Italy.

## Materials and methods

This retrospective observational cohort study was conducted from January to April 2022 in four (60 beds) tertiary referral ICU of the “Mater Domini” University Hospital and “Pugliese Ciaccio” Hospital in Catanzaro (Italy), “Annunziata” Hospital in Cosenza (Italy), and “Grande Ospedale Metropolitano” in Reggio Calabria (Italy), after local Ethical committee approval (Ethical Committee Approval number 56 on 17th February 2022). Informed consent was waived due to the retrospective observational design of the study.

We included all adult (i.e., >18 years/old) patients admitted to ICU for SARS-CoV-2 related pneumonia from January to April 2022. SARS-CoV-2 infection was ascertained through polymerases chain reaction nasal swab.

Clinical records were screened and data collected on a dedicated and customized database on Microsoft Excel. For every patient, we collected the demographic characteristics, the presence of comorbidities and the vaccination status (no doses, incomplete first-cycle, complete first cycle, and “booster” dose). The cohort of patients was therefore stratified into unvaccinated (no doses or incomplete first-cycle) and vaccinated (complete first-cycle or “booster” doses) patients.

We also collected the use of High-Flow through Nasal Cannula (HFNC), Continuous Positive Airway Pressure (CPAP) and/or Non-Invasive Ventilation (NIV) before ICU admission.

High-Flow through Nasal Cannula was considered failed and need to start NIV or intubation if the patient experienced severe dyspnea with signs of increased respiratory effort and use of accessory respiratory muscles, respiratory rate over 30 breaths/min, arterial partial pressure (PaO_2_) to inspired oxygen fraction ratio (PaO_2_/FiO_2_) <200, pH under 7.34 (17). As previously published (18), CPAP and NIV failure (followed by intubation) was defined by the presence of 1 major or two minor criteria for ≥1 h. Major criteria were: respiratory arrest; respiratory pause with unconsciousness; severe hemodynamic instability (i.e., systolic blood pressure <90 mmHg despite an adequate volume resuscitation); intolerance to all CPAP/NIV interfaces leading to treatment discontinuation. Minor criteria were: worsening of PaO_2_/FiO_2_ ≥ 30% from baseline; PaO_2_/FiO_2_ < 100; 20% increase of arterial partial pressure of carbon dioxide (PaCO_2_); worsening of alertness; new onset or persistent respiratory distress; and exhaustion (18).

During the ICU length of stay, we recorded the rate of patients undergoing intubation and invasive mechanical ventilation (iMV) and the rate of patients requiring prone positioning, neuromuscular block or Extra-Corporeal Membrane Oxygenation (ECMO).

In addition, we collected the following data at ICU admission, and 24 h and 7 days after ICU admission: (1) mode of respiratory support, positive end-expiratory pressure (PEEP) and inspiratory oxygen fraction (FiO_2_); and (2) arterial blood gases (ABGs).

Weaning from iMV was conducted according to previously described criteria (19–22). In particular, patients were switched from volume controlled to pressure support ventilation once able to trigger the ventilator. The FiO_2_ and PEEP were set to maintain peripheral oxygen saturation (SpO_2_) between 92 and 96%, while the inspiratory pressure support was titrated to generate a tidal volume of 6–8 ml/kg of ideal body weight. Once ready, a low (2 cm/H_2_O) PEEP spontaneous breathing trial was conducted for 30 min. Patients succeeding the spontaneous breathing trial (19–22) were immediately extubated. If the patient was considered at risk for post-extubation respiratory failure, non-invasive ventilation was applied through a mask or helmet for the next 12 h (23). If post-extubation respiratory failure ensued, reintubation was immediately performed (23). HFNC were also suggested over standard oxygen therapy after extubation (22, 24, 25).

Noteworthy, ventilator was set to guarantee a protective mechanical ventilation, i.e., a tidal volume of 6 ml*kg of predicted body weight, a plateau pressure < 30 cmH_2_O and a driving pressure < 15 cmH_2_O (26). Finally, we recorded the number of patients requiring tracheostomy, the ICU length of stay and mortality for the whole population and separately for unvaccinated and vaccinated patients.

### Statistical analysis

No statistical sample size calculation was performed *a priori*, and the sample size was equal to the number of patients treated in the participating ICUs during the study period. The normal distribution of data was assessed with the Kolmogorov–Smirnov test. Non-parametric data were expressed as median (25–75th interquartile range), whereas parametric data as mean (standard deviation). Categorical data were expressed as count and percentage and compared with the χ-square test. Continuous data were compared with the Mann–Whitney U-test or Student *t*-test, as indicated. Mean differences (95% confidence intervals) were also displayed for the statistically significant results. *P*-values < 0.05 were considered statistically significant for all tests.

## Results

A total of 272 patients were admitted to ICUs of Calabria region during the study period. Demographic characteristics and comorbidities are listed in Table 1. Of note, vaccinated patients were older and with a lower weight, as compared to unvaccinated cohort (see Table 1).

**TABLE 1:** Demographic characteristics and comorbidities.

	Patients	Unvaccinated	Vaccinated	*P*-value
	(*n* = 272)	(*n* = 161)	(*n* = 111)	
Age (years)	65 (14)	63 (16)	67 (11)	0.012
Male Gender–*n* (%)	174(64%)	102(63.4%)	72(64.8%)	0.799
Weight (kg)	80 (20)	84 (20)	78 (15)	0.014
Height (cm)	169 (8)	170 (8)	169 (7)	0.098
Body mass index (kg/m^2^)	28.2 (7.2)	28.9 (7.0)	27.2 (4.6)	0.085
Smoker–*n* (%)	64(23.5%)	36(22.4%)	28(25.2%)	0.299
Alcohol abuse– *n* (%)	5(1.8%)	4(2.5%)	1(0.9%)	0.339
**Comorbidities–*n* (%)**				
*Chronic respiratory disease*	42(15.4%)	24(14.9%)	18(16.2%)	0.769
*Arterial hypertension*	154(56.6%)	84(52.2%)	70(63.1%)	0.075
*Cardiovascular disease*	85(31.3%)	44(27.3%)	41(36.9%)	0.093
*Diabetes*	81(29.8%)	48(29.8%)	33(29.7%)	0.988
*Chronic renal failure*	37(13.6%)	12(7.5%)	25(22.5%)	<0.001
*Chronic liver disease*	8(2.9%)	3(1.9%)	5(4.5%)	0.205
*Autoimmune disease*	18(6.6%)	4(2.5%)	14(12.6%)	0.001
*Malignancy*	32(11.8%)	4(2.5%)	28(25.2%)	<0.001

In our cohort of patients, 153 (57%) received no doses of vaccine against SARS-CoV-2, 8 (3%) an incomplete first-cycle vaccination, 42 (15%) were vaccinated with the complete first-cycle vaccination and 69 (25%) already received the “booster” dose. Worth mentioning, among the 111 (complete first cycle and “booster”) vaccinated patients, 42 were taking immunosuppressive drugs or chronic corticosteroids therapy at home and 28 were affected by an active malignancy. On the opposite, among 161 unvaccinated patients, 8 (χ^2^ = 47.311, *p* < 0.001) were taking immunosuppressive drugs or chronic corticosteroids therapy at home and 4 (χ^2^ = 32.731, *p* < 0.001) were affected by an active malignancy. Finally, all vaccinated patients received the last vaccine dose at least 5 months before ICU admission.

### Respiratory support

Fifty-eight (21.3%) patients received HFNC for a median time of 3 (2, 5) days, whereas 111 (40.8%) underwent to CPAP, or NIV for a median time of 4 (2, 7) days before ICU admission. Sixty-seven (24.6%) patients failed HFNC, CPAP, or NIV, requiring intubation and iMV. Eighty-nine (32.7%) patients were intubated in the emergency department and transferred to the ICU. Therefore, 156 (57.3%) patients received iMV at ICU admission (Table 2). One-hundred ten patients required neuromuscular block, 42 patients were prone-positioned and 7 patients received veno-venous ECMO.

**TABLE 2:** Ventilatory support and gas exchange at Intensive Care Units (ICU) admission, 24 h and 7 days after.

	Overall population	Unvaccinated patients	Vaccinated patients		*P*-value
			
Respiratory support	*n* = 272	*n* = 161	*n* = 111		
**ICU admission**					
*HFNC n (%*)	14 (5.2%)	8 (5.0%)	6 (5.4%)	χ^2^ = 0.026	0.873
*CPAP/NIV n (%*)	102 (37.5%)	54 (33.5%)	48 (43.3%)	χ^2^ = 2.639	0.104
*iMV n (%*)	156 (57.3%)	99 (61.5%)	57 (51.3%)	χ^2^ = 2.672	0.097
**Gas Exchange**					
pH	7.44 (7.41; 7.47)	7.44 (7.42; 7.47)	7.44 (7.42; 7.47)		0.973
PaCO_2_ (mmHg)	39.3 (36.0; 45.0)	39.7 (36.3; 45.4)	39.0 (34.6; 44.2)		0.090
PaO_2_/FiO_2_ (mmHg)	190 (158; 217)	182 (150; 215)	196 (168; 220)	MD: 17, 95% CI (7–27)	0.005

**Respiratory support**	***n*** ** = 270**	***n*** ** = 159**	***n*** ** = 111**		

***24 h after ICU admission***					
*HFNC n (%*)	3 (1.1%)	2 (1.3%)	1 (0.9%)	χ^2^ = 0.076	0.783
*CPAP/NIV n (%*)	113 (41.9%)	60 (37.7%)	53 (47.7%)	χ^2^ = 2.692	0.101
*iMV n (%*)	154 (57%)	97 (61.0%)	57 (51.4%)	χ^2^ = 2.487	0.115
**Gas exchange**					
pH	7.40 (7.39; 7.42)	7.41 (7.39; 7.42)	7.40 (7.40; 7.42)		0.356
PaCO_2_ (mmHg)	41.2 (38.6; 44.3)	41.8 (38.9; 44.9)	40.8 (38.7; 44.2)		0.156
PaO_2_/FiO_2_ (mmHg)	198 (165; 223)	183 (149; 211)	211 (192; 233)	MD: 30, 95% CI (21–40)	<0.001

**Respiratory support**	***n*** ** = 190**	***n*** ** = 122**	***n*** ** = 68**		

***7 days after ICU admission***					
*HFNC n (%*)	47 (24.7%)	23 (18.9%)	24 (35.3%)	χ^2^ = 6.340	0.012
*CPAP/NIV n (%*)	46 (24.2%)	35 (28.7%)	11 (16.2%)	χ^2^ = 3.725	0.054
*iMV n (%*)	97 (51.1%)	64 (52.5%)	33 (48.5%)	χ^2^ = 0.270	0.604
**Gas exchange**					
pH	7.40 (7.39; 7.41)	7.40 (7.39; 7.41)	7.40 (7.39; 7.41)		0.891
PaCO_2_ (mmHg)	41.8 (40.1; 43.2)	41.8 (40.2; 43.2)	41.2 (39.4; 43.4)		0.343
PaO_2_/FiO_2_ (mmHg)	219 (171; 246)	211 (166; 240)	239 (196; 259)	MD: 20, 95% CI (6–35)	0.001

HFNC, High-Flow Nasal Cannula; FiO_2_, inspired fraction of oxygen; PaCO_2_, arterial partial pressure of carbon dioxide; PaO_2_, arterial partial pressure of oxygen; PaO_2_/FiO_2_, ratio between PaO_2_and FiO_2_; CPAP, Continuous Positive Airway Pressure; NIV, Non-Invasive Ventilation; PEEP, Positive End-Expiratory Pressure; iMV, invasive Mechanical Ventilation; MD, Mean Difference; 95% CI, 95% confidence interval.

By analyzing separately unvaccinated and vaccinated patients, we recorded that 99 out of 161 unvaccinated patients underwent iMV, whereas 57 out of 111 in the vaccinated cohort (61.5% vs. 51.3%; χ^2^ = 2.672, *p* = 0.097) (Table 2). Among patients undergoing iMV, 69 unvaccinated patients and 41 vaccinated patients received neuromuscular block (69.7% vs. 71.9%; χ^2^ = 0.087, *p* = 0.768). Finally, 33 unvaccinated patients and 9 vaccinated patients were also prone-positioned (33.3% vs. 15.7%; χ^2^ = 8.409, *p* = 0.004). All patients receiving ECMO were unvaccinated.

Table 2 lists the respiratory support and ABGs at ICU admission, at 24 h and 7 days after admission in the overall population and separately in the unvaccinated and vaccinated populations. As compared to unvaccinated patients, vaccinated patients were characterized by a better oxygenation (i.e., PaO_2_/FiO_2_) throughout the whole ICU length of stay.

Detailed data on gas exchange in unvaccinated and vaccinated patients stratified per type of respiratory support are shown in Supplementary Table 1 (ICU Admission), Supplementary Table 2 (24 h after ICU Admission), and Supplementary Table 3 (7 days after ICU Admission).

### Clinical outcomes

A total of 17 patients required percutaneous tracheostomy for prolonged weaning from iMV; in particular, 14 (7%) unvaccinated and 3 (3%) vaccinated patients were tracheostomized during the ICU length of stay (χ^2^ = 4.027, *p* = 0.045).

The overall ICU length of stay was 11.4 (±7.1) days. Unvaccinated patients required ICU cares for a period of 12.2 (±7.3) days, whereas ICU length of stay was 10.4 (±6.7) days in vaccinated patients [MD: 1.8, 95% CI (0.1–3.5) days; *p* = 0.036].

Finally, 62 (38.5%) unvaccinated patients died during the ICU length of stay, whereas 27 (24.3%) patients in the vaccinated population (χ^2^ = 6.005, *p* = 0.014). Septic shock induced by multidrug resistant (MDR) bacteria was the leading cause of death in 26 (42%) unvaccinated patients and in 24 (89%) vaccinated patients (χ^2^ = 16.840, *p* < 0.001). All other patients died because of respiratory failure related to SARS-CoV-2 infection.

### Patients with first complete cycle of vaccination versus “booster” dose

Table 3 enlisted the demographic characteristics and comorbidities of patients vaccinated with the sole first complete vaccination cycle and with the “booster” dose. Cohorts of patients were homogeneous apart of the rate of patients with autoimmune disease that was more present in the former population (21.4% versus 7.3%; χ^2^ = 4.764, *p* < 0.029). As shown in Table 4, the type of ventilatory support and gas exchanges were similar between cohorts of patients at ICU admission, 24 h and 7 days after.

**TABLE 3:** Demographic characteristics and comorbidities–Comparison between patients with first complete cycle of vaccination versus “booster” dose.

	Vaccinated (*n* = 111)	Complete 1^st^ cycle (*n* = 42)	“Booster” dose (*n* = 69)	*P*-value
Age (years)	67 (11)	65 (11)	69 (11)	0.060
Male Gender–*n* (%)	72 (64.8%)	26 (61.9%)	46 (66.7%)	0.610
Weight (kg)	78 (15)	79 (14)	77 (16)	0.446
Height (cm)	169 (7)	169 (7)	169 (8)	0.821
Body mass index (kg/m^2^)	27.2 (4.6)	27.4 (4.5)	26.4 (4.7)	0.286
Smoker–*n* (%)	28 (25.2%)	11 (26.2%)	17 (24.7%)	0.855
Alcohol abuse–*n* (%)	1 (0.9%)	1 (2.4%)	0 (0%)	0.198
**Comorbidities–*n* (%)**				
*Chronic respiratory disease*	18 (16.2%)	7 (16.7%)	11 (15.9%)	0.920
*Arterial hypertension*	70 (63.1%)	25 (59.5%)	45 (64.3%)	0.547
*Cardiovascular disease*	41 (36.9%)	11 (31.0%)	30 (40.6%)	0.308
*Diabetes*	33 (29.7%)	10 (23.8%)	23 (33.3%)	0.287
*Chronic renal failure*	25 (22.5%)	8 (19.1%)	17 (24.6%)	0.494
*Chronic liver disease*	5 (4.5%)	1 (2.4%)	4 (5.8%)	0.400
*Autoimmune disease*	14 (12.6%)	9 (21.4%)	5 (7.3%)	0.029
*Malignancy*	28 (25.2%)	8 (19.1%)	20 (29.0%)	0.242

**TABLE 4:** Ventilatory support and gas exchange at Intensive Care Units (ICU) admission, 24 h and 7 days after between patients with first complete cycle of vaccination and “booster” dose.

	Vaccinated patients	Complete 1stcycle	“Booster” dose	*P*-value
			
Respiratory support	*n* = 111	*n* = 42	*n* = 69	
**ICU admission**				
*HFNC n (%*)	6 (5.4%)	1 (2.4%)	5 (7.2%)	0.272
*CPAP/NIV n (%*)	48 (43.3%)	17 (40.5%)	31 (44.9%)	0.124
*iMV n (%*)	57 (51.3%)	24 (57.1%)	33 (47.9%)	0.341
**Gas exchange**				
pH	7.44 (7.42; 7.47)	7.43 (7.41; 7.46)	7.44 (7.41; 7.47)	0.992
PaCO_2_ (mmHg)	39.0 (34.6; 44.2)	38.0 (33.2; 42.1)	39.4 (35.7; 45.0)	0.251
PaO_2_/FiO_2_ (mmHg)	196 (168; 220)	207 (160; 236)	193 (171; 214)	0.298
***24 h after ICU admission***				
*HFNC n (%*)	1 (0.9%)	1 (2.4%)	0 (0%)	0.198
*CPAP/NIV n (%*)	53 (47.7%)	17 (40.5%)	36 (52.1%)	0.232
*iMV n (%*)	57 (51.4%)	24 (57.1%)	33 (47.9%)	0.341
**Gas exchange**				
pH	7.40 (7.40; 7.42)	7.41 (7.39; 7.42)	7.40 (7.40; 7.42)	0.913
PaCO_2_ (mmHg)	40.8 (38.7; 44.2)	39.8 (38.1; 42.7)	41.5 (38.7; 44.3)	0.096
PaO_2_/FiO_2_ (mmHg)	211 (192; 233)	216 (190; 239)	210 (193; 228)	0.397

**Respiratory support**	***n*** ** = 68**	***n*** ** = 24**	***n*** ** = 44**	

***7 days after ICU admission***				
*HFNC n (%*)	24 (35.3%)	8 (33.3%)	16 (36.4%)	0.803
*CPAP/NIV n (%*)	11 (16.2%)	4 (16.7%)	7 (15.9%)	0.935
*iMV n (%*)	33 (48.5%)	12 (50.0%)	21 (47.7%)	0.856
**Gas exchange**				
pH	7.40 (7.39; 7.41)	7.40 (7.39; 7.41)	7.40 (7.39; 7.41)	0.981
PaCO_2_ (mmHg)	41.2 (39.4; 43.4)	41.2 (39.4; 43.5)	41.2 (39.5; 43.2)	0.947
PaO_2_/FiO_2_ (mmHg)	239 (196; 259)	225 (196; 253)	240 (194; 260)	0.501

HFNC, High-Flow Nasal Cannula; FiO_2_, inspired fraction of oxygen; PaCO_2_, arterial partial pressure of carbon dioxide; PaO_2_, arterial partial pressure of oxygen; PaO_2_/FiO_2_, ratio between PaO_2_ and FiO_2_; CPAP, Continuous Positive Airway Pressure; NIV, Non-Invasive Ventilation; PEEP, Positive End-Expiratory Pressure; iMV, invasive Mechanical Ventilation.

Remarkably, the ICU length of stay in vaccinated patients with only the complete first cycle was 8 (6, 12) days, whereas that one in patients receiving the “booster” dose was 9 (7, 11) days (*p* = 0.662). In addition, the mortality rate was 23.8 and 23.1%, respectively (*p* = 0.940).

## Discussion

Our retrospective study shows that vaccinated patients are characterized by a less severe gas change impairment during the entire ICU length of stay, lower rate of tracheostomy and ICU mortality, and shorter ICU length of stay, as compared to unvaccinated patients.

A recent cohort study conducted in a northern region of Italy reported a 18% rate of vaccinated patients admitted to the ICU, with a median time from vaccination to ICU admission of 5 to 6 months (14). Another multicenter observational study, conducted in Spain from January to September 2021, reported that only 7% of patients admitted to the ICU for severe COVID-19 was vaccinated (27). These findings are different by our results. Firstly, the study by Lorenzoni et al. (14) was conducted from May to December 2021, when only the 57.5% of the total population in Italy received at least one dose of anti-SARS-CoV-2 vaccine (28). Similarly, the Spanish study was conducted in the same period and the 69% of Spanish inhabitants were vaccinated (27). On the opposite, we collected our data after the beginning of the “booster” vaccination program, when more than 90% of inhabitants in Calabria region already received at least the first complete vaccination cycle. By considering the proportion between the two population, unvaccinated patients have a 10-fold higher incidence of ICU admission. The percentage of vaccinated Italian inhabitants mainly increased because of the extensive use of the European Digital Green Certificate, also called “Green Pass,” required by Government decrees to enter into public activities (i.e., restaurants, cinema, museum, …) from July 2021, and mandatory for the access to work from September 2021 (28). It should also be mentioned that the emergence of new SARS-CoV-2 variants may confer decreased vaccine effectiveness (29). In fact, from November 2021 the Omicron variant overtook the Delta as dominant one (30).

Despite these differences, we also report that vaccinated patients admitted to the ICU are older than unvaccinated patients and they are characterized by a spectrum of comorbidities including immunosuppression, malignancy or chronic renal disorders (27, 29, 31).

Furthermore, we also found that vaccinated patients received the last vaccine dose at least 5 months before ICU admission (14). Remarkably, no differences in demographic characteristics, comorbidities, ventilatory support, gas exchange or clinical outcomes were found between patients with the first full vaccination dose and those having received the “booster” dose. Therefore, it seems like that the “booster” dose has no beneficial effects on clinical outcomes. However, as mentioned above, patients received their last dose at least 5 months before. We may speculate that the time from the last dose, rather than the number of received doses, may play a major role in the development of severe COVID-19. It is already known that the efficacy of vaccination wanes over time (12). In addition, Bidar et al. (15) have also reported that critically ill COVID-19 patients lack of SARS-CoV-2-specific cellular response, despite an apparent effective vaccination 2 to 6 months before.

As compared to unvaccinated patients, vaccinated ones were also characterized by a slightly better gas exchange, even if the applied respiratory support was not different between cohorts in the beginning of ICU admission. In this regard, we did not find a reduced need for iMV, despite the better oxygenation in vaccinated patients. It should be noted, however, that the different gas change impairment is not large and clinically relevant, despite the statistical significance. Of note, we recorded a significantly higher rate of extubated patients within the first week ICU admission and receiving HFNC in the vaccinated group, as well as the need for tracheostomy and the ICU length of stay were lower, as compared to unvaccinated patients. Interestingly, unvaccinated patients showed a time-course of physiological variables similar to those reported in another Italian study during the first wave, although the SARS-CoV-2 variant was different (32).

We also observed a higher mortality rate in unvaccinated patients, as opposed to vaccinated ones, that was principally related to the acute respiratory failure sustained by the SARS-CoV-2 infection. The mortality rate of unvaccinated patients was similar to those reported by some Italian studies during the first outbreak of COVID-19 in 2020, when the vaccine was not available (32, 33). On the opposite, ICU mortality in vaccinated patients, although lower than in the unvaccinated population, was mainly due to the occurrence of superinfection and septic shock by MDR, rather than SARS-CoV-2 related acute respiratory failure. By a mathematical model, it is well known that the vaccination program has altered the course of COVID-19, reducing the related mortality already after the first year of vaccine program (34). Furthermore, the impact of MDR and fungal superinfections has increased from the beginning of the pandemic, severely afflicting the survival rate of critically ill COVID-19 patients (35–38).

This study has limitations. First, the retrospective nature limits the strength of our findings. Second, we have analyzed patients from a restricted region with less than 2 million inhabitants, in a close time span. Therefore, our findings may be different in other settings. Third, we could not collect data about the type of vaccine administered because this information was not available, a limitation that we share with the study by Lorenzoni et al. (14). Fourth, it should be interesting to assess if patients with higher rates of intubation had active malignancy or were otherwise immunosuppressed and if septic shock was the leading cause of death (especially in vaccinated patients) to know whether they were affected by malignancy or immunosuppressed. However, given the low number of patients with these characteristics, a specific statistical analysis would be very weak and definitive conclusions cannot be drawn. Dedicated specific trials and studies with a greater sample should be therefore designed.

Last, we included all patients with SARS-CoV-2 related pneumonia, irrespective of the presence of comorbidities. Some comorbidities are well known to increase the risk of need for iMV or death (33). However, despite the incidence of some comorbidities was higher in vaccinated than unvaccinated patients, the outcomes were better in the former group. In addition, this limitation should also be considered as possible study bias generating confounders.

In conclusion, although vaccinated patients may require ICU admission for COVID-19, their gas exchange and clinical outcomes are largely better than unvaccinated patients. No differences however, have been recorded between patients vaccinated with the sole first cycle vaccination or “booster.” Further studies should be conducted in severe COVID-19 patients, that received the last dose of vaccine within the last 5 months, to describe their clinical features and outcomes.

## Data availability statement

The raw data supporting the conclusions of this article will be made available by the authors, without undue reservation.

## Ethics statement

The studies involving human participants were reviewed and approved by Calabria Centro Ethical Committee, Catanzaro, Italy. Approval number: 56 on 17th February 2022. Written informed consent for participation was not required for this study in accordance with the national legislation and the institutional requirements.

## Author contributions

AB, FL, and EG were responsible for conception and design of the study, and the acquisition, analysis and interpretation of the data, and for drafting and revising the article for final approval of the version to be published. SM, EB, PP, GN and MG were responsible for the acquisition, analysis and interpretation of the data, and for revising the article for final approval of the version to be published. EG was responsible for conception and design of the study, and the acquisition, analysis and interpretation of the data, and for drafting and revising the article for final approval of the version to be published. All authors contributed to the article and approved the submitted version.
