# Supplementary material for: Characteristics of unvaccinated and vaccinated critically ill COVID-19 patients in calabria region (Italy): A retrospective study
Source: Front Med (Lausanne). 2022 Nov 24;9:1042411. doi: 10.3389/fmed.2022.1042411 (PMC9729689; doi:10.3389/fmed.2022.1042411)
Supplement: Supplementary file 1 [file Data_Sheet_1.docx]

**SUPPLEMENTARY MATERIAL**

- **Table S1 – Gas Exchange in the overall population and in unvaccinated and vaccinated patients at ICU Admission, stratified per type of respiratory support**
- **Table S2 – Gas Exchange in the overall population and in unvaccinated and vaccinated patients 24 hours after ICU Admission, stratified per type of respiratory support**
- **Table S3 – Gas Exchange in the overall population and in unvaccinated and vaccinated patients 7 days after ICU Admission, stratified per type of respiratory support**

**List of abbreviations:**

HFNC, High-Flow Nasal Cannula; FiO_2_, inspired fraction of oxygen; PaCO_2_, arterial partial pressure of carbon dioxide; PaO_2_, arterial partial pressure of oxygen; PaO_2_/FiO_2_, ratio between PaO_2_ and FiO_2_; CPAP, Continuous Positive Airway Pressure; NIV, Non-Invasive Ventilation; PEEP, Positive End-Expiratory Pressure; iMV, invasive Mechanical Ventilation; MD, Mean Difference; 95% CI, 95% confidence interval.

**Table S1 – Gas Exchange in the overall population and in unvaccinated and vaccinated patients at ICU Admission, stratified per type of respiratory support**

|  | **Overall population**  **(n=272)** | **Unvaccinated patients**  **(n=161)** | **Vaccinated patients**  **(n=111)** |  | **P value** |
| --- | --- | --- | --- | --- | --- |
| **HFNC n (%)** | **14 (5.2%)** | **8 (5.0%)** | **6 (5.4%)** | χ^2^= 0.026 | 0.873 |
| FiO_2_ (%) | 40 [35; 40] | 38 [35; 40] | 40 [36; 44] |  | 0.335 |
|  |  |  |  |  |  |
| ***Gas Exchange*** |  |  |  |  |  |
| pH | 7.44 [7.39;7.46] | 7.43 [7.38; 7.48] | 7.44 [7.41; 7.46] |  | 0.846 |
| PaCO_2_ (mmHg) | 42.0 [36.9; 48.7] | 45.0 [40.2; 51.0] | 38.7 [31.5; 49.0] |  | 0.245 |
| PaO_2_/FiO_2_ (mmHg) | 192 [181; 215] | 186 [178; 211] | 194 [189; 256] |  | 0.228 |
|  |  |  |  |  |  |
| **CPAP/NIV n (%)** | **102 (37.5%)** | **54 (33.5%)** | **48 (43.3%)** | χ^2^= 2.639 | 0.104 |
| PEEP (cmH_2_O) | 8 [7; 8] | 8 [7; 8] | 8 [7; 8] |  | 0.496 |
| FiO_2_ (%) | 40 [35; 40] | 40 [35; 40] | 40 [35; 40] |  | 0.799 |
|  |  |  |  |  |  |
| ***Gas Exchange*** |  |  |  |  |  |
| pH | 7.45 [7.42; 7.47] | 7.45 [7.42; 7.47] | 7.44 [7.41; 7.47] |  | 0.984 |
| PaCO_2_ (mmHg) | 40.2 [36.2; 45.0] | 39.9 [36.8; 45.2] | 40.5 [34.1; 44.7] |  | 0.400 |
| PaO_2_/FiO_2_ (mmHg) | 214 [192; 234] | 214 [196; 232] | 213 [189; 238] |  | 0.973 |
|  |  |  |  |  |  |
| **iMV n (%)** | **156 (57.3%)** | **99 (61.5%)** | **57 (51.3%)** | χ^2^= 2.672 | 0.097 |
| PEEP (cmH_2_O) | 8 [8; 10] | 9 [8; 10] | 8 [7; 10] |  | 0.062 |
| FiO_2_ (%) | 50 [45; 55] | 50 [45; 60] | 45 [40; 50] | MD: 0.7, 95%CI [0.1 - 1.4] | 0.002 |
|  |  |  |  |  |  |
| ***Gas Exchange*** |  |  |  |  |  |
| pH | 7.44 [7.41; 7.47] | 7.44 [7.41; 7.47] | 7.43 [7.40; 7.48] |  | 0.918 |
| PaCO_2_ (mmHg) | 39.0 [35.6; 44.2] | 39.0 [35.3; 45.1] | 38.0 [35.9; 42.5] |  | 0.246 |
| PaO_2_/FiO_2_ (mmHg) | 165 [139; 196] | 161 [133; 188] | 184 [148; 207] | MD: 19, 95%CI [6 - 32] | 0.006 |
|  |  |  |  |  |  |

**Table S2 – Gas Exchange in the overall population and in unvaccinated and vaccinated patients 24 hours after ICU Admission, stratified per type of respiratory support**

|  | **Overall population**  **(n=270)** | **Unvaccinated patients**  **(n=159)** | **Vaccinated patients**  **(n=111)** |  | **P value** |
| --- | --- | --- | --- | --- | --- |
| **HFNC n (%)** | ***3 (1.1%)*** | ***2 (1.3%)*** | ***1 (0.9%)*** | *χ^2^= 0.076* | *0.783* |
| *FiO_2_ (%)* | 30 [30; 35] | 33 [30; 35] | 35 [35; 35] |  | n.a. * |
|  |  |  |  |  |  |
| ***Gas Exchange*** |  |  |  |  |  |
| pH | 7.42 [7.40; 7.42] | 7.42 [7.42; 7.42] | 7.40 [7.40; 7.40] |  | n.a. * |
| PaCO_2_ (mmHg) | 42.3 [39.7; 50.1] | 46.2 [42.3; 50.1] | 39.7 [39.7; 39.7] |  | n.a. * |
| PaO_2_/FiO_2_ (mmHg) | 253 [234; 253] | 244 [234; 253] | 253 [253; 253] |  | n.a. * |
|  |  |  |  |  |  |
| **CPAP/NIV n (%)** | ***113 (41.9%)*** | ***60 (37.7%)*** | ***53 (47.7%)*** | *χ^2^= 2.692* | *0.101* |
| *PEEP (cmH_2_O)* | 8 [7; 8] | 8 [8; 10] | 7 [5; 8] | MD: 2, 95%CI [1 - 2] | <0.001 |
| *FiO_2_ (%)* | 40 [35; 40] | 40 [35; 40] | 35 [35; 40] | MD: 1, 95%CI [0 - 3] | 0.015 |
|  |  |  |  |  |  |
| ***Gas Exchange*** |  |  |  |  |  |
| pH | 7.41 [7.40; 7.43] | 7.42 [7.39; 7.44] | 7.41 [7.40; 7.43] |  | 0.158 |
| PaCO_2_ (mmHg) | 41.8 [38.8; 45.5] | 41.8 [39.3; 46.7] | 41.2 [38.6; 45.0] |  | 0.282 |
| PaO_2_/FiO_2_ (mmHg) | 214 [197; 234] | 210 [192; 222] | 223 [210; 245] | MD: 16, 95%CI [7 - 26] | <0.001 |
|  |  |  |  |  |  |
| **iMV n (%)** | ***154 (57%)*** | ***97 (61.0%)*** | ***57 (51.4%)*** | *χ^2^= 2.487* | *0.115* |
| *PEEP (cmH_2_O)* | 10 [8; 10] | 10 [8; 10] | 8 [7; 10] | MD: 1, 95%CI [1 - 2] | <0.001 |
| *FiO_2_ (%)* | 45 [40; 55] | 50 [45; 60] | 40 [40; 45] | MD: 9, 95%CI [5 - 12] | <0.001 |
|  |  |  |  |  |  |
| ***Gas Exchange*** |  |  |  |  |  |
| pH | 7.40 [7.39; 7.42 | 7.40 [7.39; 7.42] | 7.40 [7.40; 7.42] |  | 0.882 |
| PaCO_2_ (mmHg) | 41.5 [38.6; 44.2] | 41.6 [38.2; 44.4] | 40.5 [38.7; 43.5] |  | 0.423 |
| PaO_2_/FiO_2_ (mmHg) | 175 [144; 205] | 162 [133; 188] | 203 [175; 216] | MD: 35, 95%CI [23 - 47] | <0.001 |
|  |  |  |  |  |  |

* comparisons between not-vaccinated and vaccinated patients cannot be done due to the single value in vaccinated group

**Table S3 – Gas Exchange in the overall population and in unvaccinated and vaccinated patients 7 days after ICU Admission, stratified per type of respiratory support**

|  | **Overall population**  **(n=190)** | **Unvaccinated patients**  **(n=122)** | **Vaccinated patients**  **(n=68)** |  | **P value** |
| --- | --- | --- | --- | --- | --- |
| **HFNC n (%)** | **47 (24.7%)** | **23 (18.9%)** | **24 (35.3%)** | χ^2^= 6.340 | 0.012 |
| FiO_2_ (%) | 35 [30; 35] | 35 [30; 35] | 35 [31; 35] |  | 0.416 |
|  |  |  |  |  |  |
| ***Gas Exchange*** |  |  |  |  |  |
| pH | 7.40 [7.39; 7.40] | 7.40 [7.39; 7.42] | 7.40 [7.39; 7.40] |  | 0.346 |
| PaCO_2_ (mmHg) | 41.2 [40.2; 43.1] | 41.2 [40.3; 43.1] | 41.1 [39.2; 43.2] |  | 0.602 |
| PaO_2_/FiO_2_ (mmHg) | 251 [240; 266] | 243 [228; 263] | 257 [246; 269] | MD: 12, 95%CI [1 - 26] | 0.038 |
|  |  |  |  |  |  |
| **CPAP/NIV n (%)** | **46 (24.2%)** | **35 (28.7%)** | **11 (16.2%)** | χ^2^= 3.725 | 0.054 |
| PEEP (cmH_2_O) | 7 [7; 8] | 8 [7; 8] | 6 [5; 8] |  | 0.052 |
| FiO_2_ (%) | 35 [35; 40] | 35 [35; 40] | 35 [35; 40] |  | 0.817 |
|  |  |  |  |  |  |
| ***Gas Exchange*** |  |  |  |  |  |
| pH | 7.40 [7.40; 7.42] | 7.40 [7.40; 7.42] | 7.41 [7.40; 7.42] |  | 0.570 |
| PaCO_2_ (mmHg) | 39.8 [41.9; 44.9] | 42.0 [39.9; 44.7] | 41.1 [38.2; 46.1] |  | 0.471 |
| PaO_2_/FiO_2_ (mmHg) | 239 [219; 247] | 237 [217; 246] | 240 [220; 251] |  | 0.699 |
|  |  |  |  |  |  |
| **iMV n (%)** | **97 (51.1%)** | **64 (52.5%)** | **33 (48.5%)** | χ^2^= 0.270 | 0.604 |
| *PEEP (cmH_2_O)* | 10 [8; 10] | 10 [8; 10] | 8 [7; 10] | MD: 1, 95%CI [0 - 2] | 0.012 |
| *FiO_2_ (%)* | 45 [40; 53] | 45 [40; 53] | 40 [35; 53] | MD: 4, 95%CI [1 - 8] | 0.023 |
|  |  |  |  |  |  |
| ***Gas Exchange*** |  |  |  |  |  |
| pH | 7.40 [7.38; 7.41] | 7.39 [7.37; 7.40] | 7. 40 [7.38; 7.41] |  | 0.388 |
| PaCO_2_ (mmHg) | 41.8 [40.2; 43.8] | 42.0 [40.1; 44.3] | 41.2 [40.2; 43.4] |  | 0.764 |
| PaO_2_/FiO_2_ (mmHg) | 171 [142; 209] | 168 [141; 195] | 195 [149; 223] | MD: 22, 95%CI [3 - 41] | 0.038 |
|  |  |  |  |  |  |
